# Selective recruitment designs for improving observational studies using electronic health records

**DOI:** 10.1002/sim.8556

**Published:** 2020-06-10

**Authors:** James E. Barrett, Aylin Cakiroglu, Catey Bunce, Anoop Shah, Spiros Denaxas

**Affiliations:** ^1^ Cancer Cell Biology and Imaging King's College London London UK; ^2^ The Francis Crick Institute London UK; ^3^ Division of Health and Social Care Research King's College London London UK; ^4^ UCL Institute of Health Informatics University College London London UK; ^5^ Health Data Research U.K. London UK; ^6^ University College London Hospitals NHS Trust London UK

**Keywords:** electronic health records, observational study, optimal experimental design, selective recruitment

## Abstract

Large‐scale electronic health records (EHRs) present an opportunity to quickly identify suitable individuals in order to directly invite them to participate in an observational study. EHRs can contain data from millions of individuals, raising the question of how to optimally select a cohort of size *n* from a larger pool of size *N*. In this article, we propose a simple selective recruitment protocol that selects a cohort in which covariates of interest tend to have a uniform distribution. We show that selectively recruited cohorts potentially offer greater statistical power and more accurate parameter estimates than randomly selected cohorts. Our protocol can be applied to studies with multiple categorical and continuous covariates. We apply our protocol to a numerically simulated prospective observational study using an EHR database of stable acute coronary disease patients from 82 089 individuals in the U.K. Selective recruitment designs require a smaller sample size, leading to more efficient and cost‐effective studies.

## 1 INTRODUCTION

Large‐scale electronic health records present the possibility of conducting prospective observational studies by directly identifying individuals that meet pre‐specified criteria.^1^, ^2^ EHRs typically contain clinical covariates and phenotypes that can be linked to laboratory tests, primary and secondary care records, as well as molecular data. In a conventional observational study, investigators typically wait for potential recruits to arrive at designated study centers—a process that can take years to complete, if at all.^3^ EHRs may potentially contain millions of patients and in many cases there will be an abundance of eligible patients for a particular study. EHRs offer the obvious advantages of faster recruitment and reduced costs but they also raise the interesting question of how to optimally select a cohort of *n* individuals from a pool of size *N* where *n*≪*N*.

The aim of an observational study is to establish a statistical relationship between covariates and clinical outcomes of interest. We assume that the covariates of interest are available in the EHR database, but that the outcomes are not, either because they are not routinely recorded or because more detailed or rigorous measurements are required. EHRs present an opportunity to select patients on the basis of their covariates in order to invite them to participate in the study. The simplest selection strategy is to randomly select *n* individuals from the pool. As we shall see this generally would not provide the greatest statistical power. An alternative strategy is to preferentially select a more “informative” cohort, where informativeness is defined in terms of covariate values. In this article, we propose a simple strategy that attempts to form a cohort in which each covariate has a uniform distribution (or approximately uniform in the case of a continuous covariate, as described below). Each member of the pool is assigned a recruitment probability. Individuals that will contribute to a uniform cohort distribution are deemed more informative, and consequently will have a higher probability of recruitment. Note that the purpose of our protocol is not to retain representativeness of the pool but rather to create a more informative cohort.

To gain some intuition for this idea, consider several patients with identical covariate values compared to several patients with slightly different covariate values. Although both groups are informative, the latter patients are inherently more informative because they tell us how the outcome depends on different values of the covariates. Our selective recruitment strategy means we are less likely to make repeated observations of similar individuals, and more likely to explore the covariate space efficiently. Statistical inference is based on observed regularities between covariates and outcomes. It is, therefore, advantageous to acquire observations evenly throughout the covariate space rather than a concentration of data points within a restricted region of the space.

As a further example, consider a pool population with a single binary covariate coded as +1 and −1. Selecting a cohort with an equal number of +1 and −1 observations will maximize statistical power. From a statistical perspective, there is no *a priori* justification for selecting more of one covariate value than the other, even if the covariate is unequally distributed in the population. The desire for an a priori uniform covariate distribution in our cohort reflects Keynes' *principle of indifference* ^4^ which states that “equal probabilities must be assigned to each of several arguments if there is an absence of positive ground for assigning unequal ones.”

The ability to be selective about which patients to invite onto a study is only possible with the emergence of large‐scale EHRs. While the clinical utility of EHRs is increasingly recognized,^5^, ^6^, ^7^, ^8^ the underlying infrastructure is still developing and the use of EHRs for research purposes is fraught with issues such as missing and incomplete data, data quality, accuracy, confidentiality, interoperability, security, and patient consent. These problems have been discussed in depth in the literature,^5^, ^6^, ^8^, ^9^ and we will restrict our focus to statistical issues relating to the use of EHRs as a recruitment aid. An example of EHR based recruitment is the European Electronic Health Record systems for Clinical Research (EHR4CR) platform.^10^

The remainder of this article is organized as follows. In Section 2, we review previous work on controlling the distribution of covariates in a clinical study. We describe our selective recruitment protocol in Section 3. In Section 4, we perform numerical simulations and study the operating characteristics of our protocol in comparison to randomized selection strategies. In Section 5, as a proof of concept, we apply our protocol to a numerically simulated observational study based on EHR data from 82 089 patients with stable acute coronary disease in the U.K. We discuss our findings in Section 6 and present our conclusions in Section 7.

## 2 BACKGROUND

The central idea behind our proposed method is to select samples on the basis of their covariate values instead of random selection. The concept of controlling the covariate distribution within a study cohort has previously been implemented in a variety of contexts. These techniques share a common theme: creating a favorable distribution of covariates in order to increase statistical power and reduce the risk of bias. The most straightforward approach is *stratified sampling* in which the population is divided into distinct strata, out of which individuals are randomly sampled.^11^ This ensures distinct subpopulations are equally represented. *Matching* is a technique that can be applied retrospectively to observational datasets containing an *exposure* (or treatment) group and a *control* group.^12^ A subset of the data is selected as a control group such that the distribution of covariates within the exposure and control group is as similar as possible. Both groups are, therefore, more comparable and estimates of group differences are less prone to bias.

When the exposure and control groups do not match perfectly, a parametric model can be used to account for differences in covariates.^13^ When there are a large number of covariates, it becomes difficult to form a matching cohort and instead *propensity score matching* can be used.^14^ Matching methods can be viewed as a means to reduce model dependent bias.^15^ This is because the parametric model used to adjust for covariate imbalances may be misspecified in practice and with matched groups, the dependence on model assumptions is diminished. All matching methods are prone to bias when unmeasured covariates are associated with the outcome of interest and it is frequently assumed that all relevant covariates are measured (although this is impossible to verify in reality).

In *two‐phase sampling* (or double sampling), auxiliary variables are measured in a sample drawn randomly from the population. It is assumed that the auxiliary variables are relatively inexpensive to measure. The primary variable of interest, assumed to be comparatively expensive, is subsequently measured in a subset of the initial sample. In ratio estimation, a two‐phase strategy can be used to estimate the mean of a certain quantity in the population and subsampling fractions can be chosen to minimize the variance of the estimators.^16^ When two‐phase sampling is used for stratification, the initial sample is divided into strata followed by stratified random sampling. In the context of this article, the EHR would represent the initial sample and the auxiliary variables would correspond to the covariates. The outcome of interest would subsequently be measured on a smaller cohort selected from the EHR pool. Applied to a categorical covariate, our proposed selective recruitment protocol is equivalent to two‐phase stratified sampling, but we additionally consider an arbitrary combination of categorical and continuous covariates.

Covariate balancing methods have also been used in the theory of experimental design. *Stratified blocking* designs randomize treatment and controls within predefined strata,^17^ thus ensuring both treatment and control groups are similar in terms of the stratified covariates. Covariate‐adaptive clinical trials allocate patients onto treatment arms in a manner that tries to minimize the covariate imbalance between arms.^18^, ^19^, ^20^ Another field that uses covariate information to select samples is *active machine learning*. The aim is to actively seek data points that are anticipated to be informative. There are various ways to define informativeness.^21^ For example, individuals that are expected to reduce the posterior entropy or reduce future prediction errors are deemed more informative. Several of these concepts were previously applied to selective recruitment trial designs.^22^

All of the above methods share the common theme of selecting samples on the basis of their covariate values, either for allocation into different treatment groups (in the context of a trial) or inclusion in a study (in the case of matching or active machine learning). Our proposed method shares this methodological theme of selecting samples according to their covariate values. Our aim is to select samples with “informative” covariate values from EHR databases for the purpose of a subsequent observational study. The aim in such an observational study is to establish statistical associations between covariates and outcomes of interest. For example, in our proof on concept in Section 5, we establish associations between various clinical and epidemiological factors and time‐to‐death (all‐cause mortality) using a Cox proportional hazards model. Our overall objective is to infer the parameters of this model and our proposal is that by selecting a cohort with uniform covariate distributions (or close to uniform), we can achieve greater statistical power. There are no treatment/exposure and control groups, and so our aim is simply to achieve a cohort in which covariates are uniformly distributed. This is in contrast to matching in which the covariate distribution of the control group is selected to be as similar as possible to the treatment/exposure group. Note that the population of interest is defined by the EHR, and in the case of our example corresponds to patients with stable coronary artery disease.

## 3 METHODS

We assume that each individual in the pool is characterized by a *d*‐dimensional vector of covariates **x**, and denote the clinical outcome of interest as *y*. We will consider both binary and time‐to‐event outcomes in this article. It is further assumed that *y* is unavailable in the EHR system, either because it is not routinely measured or requires further measurements. In this article, we will focus on selecting a cohort for a prospective observational study in which the goal is to establish the statistical relationship between **x** and *y*.

Our goal is to select a subset of *n* individuals from within a larger pool of *N* individuals. The vector **x** consists of either categorical or continuous covariates. We denote binary clinical outcomes by *y*∈{−1,+1}. Our strategy is to select individuals such that the distribution of covariates across the cohort is as close to uniform as possible. Define *r* such that *r*=1 and *r*=0 indicates whether an individual was recruited or not, and let **x**_*i*_=[*x*_*i*1_,…,*x*_*id*_] denote one realization of the covariates (i.e. one individual) . Then our goal is to achieve

(1) $$p(x=x_{i}|r=1)=p(x=x_{j}|r=1)\;\text{for}\;i,j=1,\ldots ,n.$$

Choosing a uniform distribution to reflect the absence of prior knowledge is similar in spirit to the use of *uninformative priors* in Bayesian inference.^23^ One potential problem with uninformative priors is that they depend on how a covariate is defined. A uniform distribution over height, for instance, will not correspond to a uniform distribution over body mass index (which is based on the square of height). Some uninformative priors have been developed that are invariant to re‐parameterization of a covariate such as Jeffery's prior.^24^ For the purposes of this article, we will assume that covariates have been appropriately defined in advance and use uniform distributions to reflect a lack of prior knowledge.

### 3.1 Selective recruitment with a single binary covariate

Suppose we have a single binary covariate *x*∈{−1,+1}. We can write

(2) $$\frac{p(r=1|x)p(x)}{p(r=1)}=p(x|r=1).$$

Uniformity in our recruited cohort requires *p*(*x*=+1|*r*=1)=*p*(*x*=−1|*r*=1) which implies

(3) p(r=1|x=+1)p(x=+1)=p(r=1|x=−1)p(x=−1).

This is solved by *p*(*r*=1|*x*=+1)=*p*(*x*=−1) and *p*(*r*=1|*x*=−1)=*p*(*x*=+1). If *p* is the proportion of individuals in the pool with *x*=+1, we can therefore recruit individual *i* from the pool with probability

(4) $$\rho (x_{i})=\left\{\begin{array}{ll} (1-p)/c & \text{if}\;x_{i}=+1\\ p/c & \text{if}\;x_{i}=-1 \end{array}\right.\;\text{for}\;i=1,\ldots ,N$$

where the normalization constant is $c=\sum_{i=1}^{N}\rho (x_{i})$. This normalized inverse weighted probability recruitment strategy will ensure that on average the covariate is uniformly distributed within the cohort.

### 3.2 Selective recruitment with a single continuous covariate

**FIGURE 1:**
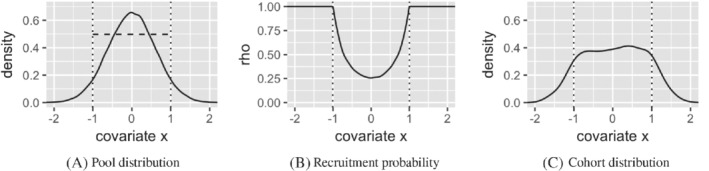
In (A) is the pool distribution (*N*=100 000) of a single covariate *x* (solid black line). The two vertical dotted lines correspond to the 0.05 and 0.95 quantiles. The horizontal dashed line corresponds to the value of *q* (as defined in Equation (5)). In (B) is the recruitment probability as a function of *x*. In (C) is the cohort distribution (*n*=1000) after selective recruitment from the pool

In the case of a continuous covariate x∈ℝ, we can write *p*(*r*=1|*x*)=*p*(*x*|*r*=1)*p*(*r*=1)/*p*(*x*). Uniformity in our cohort requires *p*(*x*|*r*=1)=*q* for a constant *q* which implies *p*(*r*=1|*x*)∝*q*/*p*(*x*). A covariate with infinite support means that selecting a uniformly distributed cohort is not possible. As a pragmatic compromise, we attempt to form a uniform cohort distribution between the 0.05 and 0.95 quantiles of the pool distribution (denoted by *x*_*l*_ and *x*_*u*_, respectively). We first generate an empirical density estimate *p*(*x*) of the pool distribution. A recruitment probability for an individual with covariate *x*_*i*_ is given by

(5) $$\rho (x_{i})=\left\{\begin{array}{ll} \frac{1}{c}\frac{q}{c^{\prime }p(x_{i})} & \text{if}\;x_{l}\leq x_{i}\leq x_{u}\\ \frac{1}{c} & \text{otherwise} \end{array}\right.\;\text{for}\;i=1,\ldots ,N$$

where *q*=1/(*x*_*u*_−*x*_*l*_). The constants *c*, defined as above, and $c^{\prime }=\max_{x_{l}\leq x\leq x_{u}}q/p(x)$ ensure the probabilities are appropriately normalized. Equation (4) is essentially a discretized version of Equation (5). An example of this can be seen in Figure 1B.

### 3.3 Selective recruitment with multiple covariates

**FIGURE 2:**
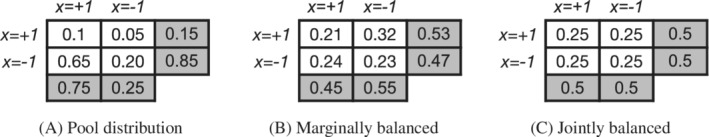
In (A) is the pool distribution of two binary covariates. In (B) is the cohort distribution after applying Equation (6) (and assuming large *N* and *n*). In (C) is a cohort with a perfectly balanced joint distribution

When we have *d* covariates, one option is to try and balance the marginal distribution of each covariate. This can be achieved by

(6) $$\rho (x_{i})=\frac{1}{c}\overset{d}{\underset{\mu =1}{\prod }}\rho_{\mu }(x_{i}),$$

where ρ_μ_(*x*_*i*_) is given by either Equation (4) or (5). An example of this protocol with two binary covariates is shown in Figure 2B. An alternative strategy when all covariates are binary is to balance the joint distribution of covariates within the cohort (as in Figure 2C). This can be achieved by simply stratifying the pool into four groups and randomly selecting the requisite number of individuals from each group. However, when the pool size is relatively small in comparison to the number of covariates, this generally would not be possible. For example, recruitment of a cohort of size *n*=100 according to Figure 2C would require 25 individuals in each stratum in the pool, which may not be possible. In these instances, the marginally balanced method may be used instead. Equation (6) is used to compute a recruitment probability for each individual in the pool. A cohort of size *n* is then obtained by using the recruitment probabilities to sample, without replacement, *n* individuals from the pool. Note that the marginally balanced method will not achieve perfectly uniform marginal distributions.

## 4 RESULTS FROM NUMERICAL SIMULATION STUDIES

In order to assess the performance of these different selection protocols, we performed several numerical simulations. We evaluated the statistical power, mean square error, and type I error rates under various conditions.

### 4.1 Binary covariates

**FIGURE 3:**
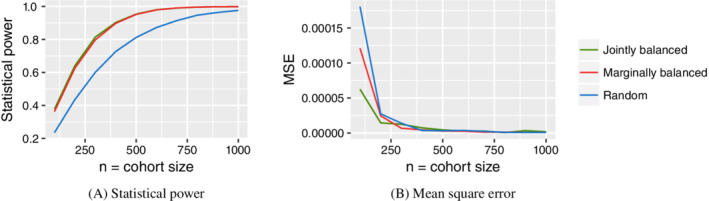
Statistical power and mean square error as a function of cohort size in the case of two binary covariates [Colour figure can be viewed at wileyonlinelibrary.com]

A pool of *N*=10 000 individuals with two binary covariates was generated from the distribution shown in Figure 2A. We recruited *n* individuals from the pool according to three different protocols, marginally balanced (Figure 2B), jointly balanced (Figure 2C), and random selection. Binary outcomes *y*=±1 were generated according to a logistic regression model *p*(*y*=+1|**x**)=1/(1+exp(−*w*_0_−**w**·**x**)) with parameters set to *w*_0_=−1/6 and **w**=(1/3,+1/3). For each cohort of size *n*, a logistic regression model was fitted and statistical power was calculated as the proportion of inferred parameters that were statistically significant at α=0.05. Statistical power and the mean square error between true and inferred parameter values as a function of cohort size *n* are plotted in Figure 3. Selective recruitment offers a clear advantage with little difference between the jointly and marginally balanced protocols. We also found that the Type I error rates in cohorts formed using the different protocols were all well controlled at the expected 5% error rate (Supplementary Figure 1). The existence of unmeasured covariate introduces a bias to the parameter estimates but this bias is independent of the cohort distribution (Supplementary Figure 2).

### 4.2 Continuous covariate

**FIGURE 4:**
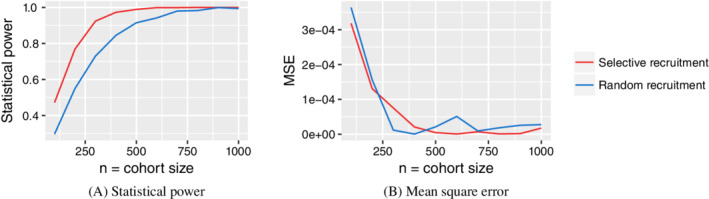
Statistical power and mean square error as a function of cohort size for the case of one continuous covariate [Colour figure can be viewed at wileyonlinelibrary.com]

A pool of *N*=10 000 individuals was generated with a single normally distributed covariate *x* with zero mean and standard deviation 0.608 (such that the 0.05 and 0.95 quantiles are equal to −1 and +1 for convenience). Cohorts were selected according to Equation (5) and compared to a randomized recruitment design. A logistic regression model with parameters *w*_0_=−1/2 and *w*=−1/4 was used to generate outcomes. The statistical power and mean square error between true and inferred parameters, obtained after fitting logistic regression models to each simulated cohort, are plotted in Figure 4. We find that the selective recruitment protocol offers a clear gain in in statistical power. For example, to achieve a power of 90%, approximately 275 individuals would need to be recruited using a selective recruitment design in comparison to approximately 500 individuals in a randomized design.

## 5 RESULTS FROM APPLICATION TO A CARDIOVASCULAR EHR DATABASE

In order to demonstrate how a selective‐recruitment protocol can be used in practice, we simulated a prospective observational study using an EHR database of 82 089 anonymized patients with stable coronary artery disease from the CALIBER resource^25^, ^26^, ^27^, ^28^ (described below). The data consist of 30 biomarkers and risk factors and the primary outcome was time‐to‐death (all‐cause mortality). Our aim was to select a cohort of *n*=1000 individuals and study the associations between the 30 covariates and time‐to‐death. We compared the operating characteristics of randomly and selectively recruited cohorts.

For the purposes of our proof‐of‐concept simulation, both covariates and the outcome of interest are already available. In practice, however, a prospective observational study would be required in situations where the desired outcome was unavailable or situations where a study with more rigorous and detailed measurements were required. In these situations, EHR resources could potentially be used for the recruitment of individuals onto a study in which the clinical outcome of interest would subsequently be measured. The type of study we are simulating is similar to the Cardiovascular Health Study which was a prospective observational study aiming to establish cardiovascular risk factors associated with 5‐year mortality in a population of 5201 adults in the United States.^29^ We propose that instead of slowly accruing 5201 individuals at designated study centers, a cohort instead could be formed using EHRs, should they be available. The results above show that a smaller (but more informative) cohort could potentially offer the same level of power as a randomly recruited cohort.

### 5.1 Data sources

CALIBER was established to provide access to longitudinal data of linked EHRs through the creation of a common data model with reproducible phenotypes and metadata. Patients were linked across three clinical data sources: the Clinical Practice Research Datalink (CPRD), Hospital Episodes Statistics (HES), and cause‐specific mortality (from the Office of National Statistics). CPRD provides information about anthropometric measurements, laboratory tests, clinical diagnoses, prescriptions, and medical procedures, coded with the Read controlled clinical terminology^30^ (which are a subset of SNOMED clinical terms). The primary care practices in CPRD and the subset of linked practices used in the present analysis are representative of the UK primary care setting and have been validated for epidemiological research.^31^, ^32^ HES provides information about diagnoses (coded with the tenth revision of the International Classification of Diseases statistical classification system) and interventional procedures related to all elective and emergency hospital admissions across all National Health Service hospitals in England.

The eligible patients were chosen from a cohort of a previous study on stable coronary artery disease prediction using CALIBER data.^33^ All variables that were chosen as predictors in the previous study were used as covariates in our simulation. These included age, diabetes, smoking, systolic blood pressure, diastolic blood pressure, total cholesterol, HDL cholesterol, serum creatinine, hemoglobin, total white blood cell count, CABG or PCI surgery within 6 months prior to study entry, abdominal aortic aneurysm prior to study entry, index of multiple deprivation (IMD), hypertension diagnosis or medication prior to study entry, use of long acting nitrates prior to study entry, diabetes diagnosis prior to study entry, peripheral arterial disease prior to study entry, and history of depression, anxiety disorder, cancer, renal disease, chronic obstructive pulmonary disease, atrial fibrillation, or stroke. We excluded the history of MI and liver disease because both were highly correlated with other covariates in our dataset. A summary of the patient population used in this study is shown in Table 1. Dichotomous covariates were coded as −1 or +1. Continuous covariates were linearly scaled such that the 0.05 and 0.95 quantiles are equal to −1 and +1, respectively. IMD and smoking were collapsed into binary variables in accordance with previous analysis of this dataset.^33^

**TABLE 1:** Summary of the full CALIBER dataset and an example of a selectively recruited cohort

Covariate	Full Caliber dataset (*N*=82 089)	Example cohort (*n*=1000)
Age	68.1 (47.0‐87.0)	73.6 (49.0‐92.0)
Male	57.2%	52.2%
Female	42.8%	47.8%
Index of multiple deprivation	8.1%	15.0%
Non‐specified coronary artery disease	1.9%	5.9%
Unstable angina	11.8%	19.7%
Non‐ST‐elevated MI	14.9%	24.5%
ST‐elevated MI	13.9%	15.6%
Coronary artery bypass graft	2.0%	7.8%
Diabetes	15.4%	34.2%
Heart failure diagnosis	9.9%	35.1%
History of arterial fibrillation	11.6%	35.8%
History of anxiety	12.0%	24.9%
History of cancer	8.0%	21.3%
History of COPD	35.1%	50.9%
History of depression	19.1%	32.1%
History of kidney disease	6.6%	28.6%
History of stroke	5.4%	20.4%
Hypertension diagnosis	88.2%	90.1%
Use of long acting nitrates	26.9%	38.7%
Peripheral arterial disease	7.0%	21.7%
Percutaneous coronary intervention	4.5%	10.6%
Smoking	55.6%	55.9%
Systolic blood pressure (mmHg)	140.5 (110‐178)	136.8 (101.5‐179)
Diastolic blood pressure (mmHg)	79.4 (60‐99.6)	75.6 (57.7‐98.5)
Serum creatinine (mol/l)	99.1 (64.8‐148.2)	111.2 (62.7‐183)
Total cholesterol (mmol/l)	5.2 (3.2‐7.8)	4.7 (2.8‐7.5)
HDL cholesterol (mmol/l)	1.4 (0.8‐2.1)	1.3 (0.7‐2.1)
Total WBC count 10^9^/l	7.4 (4.5‐11.2)	7.9 (4.4‐12.6)
Hemoglobin (g/dL)	13.7 (11.0‐16.6)	12.9 (10.2‐16.2)
Pulse (bpm)	73 (51.2‐99.9)	75.4 (51.2‐103.2)
Death	23.0%	47.5%
Censored	77.0%	52.5%

*Note*: Values are quoted to one significant figure and may not sum due to rounding. Continuous values are summarized as mean (5th‐95th percentile). Individuals with an index of multiple deprivation (IMD) score >1 were coded as +1, otherwise −1.

Abbreviations: COPD, chronic obstructive pulmonary disease; HDL, high density lipoprotein; MI, myocardial infarction; WBC, white blood cell.

Multiple imputation was implemented using multivariate imputation by chained equations in the R package mice.^34^ Imputation models were estimated separately for men and women using all 115 305 patients before exclusion criteria were applied (MI or death before study eligibility). Since many of the continuous variables were non‐normally distributed, we log‐transformed all continuous variables for imputation and exponentiated back to their original scale for analysis. Only one multiply imputed dataset was generated since any imputation errors are not expected to have a significant effect on our analyses in respect to the comparison of different designs. The distributions of observed and imputed values of all variables followed similar distributions indicating the plausibility of the imputation. Full details of covariates, study population definitions, and an overview and details of the imputation methods can be found in Section 2 of the Supplementary material.

### 5.2 Simulation of a prospective observational study using the CALIBER dataset

The pool of available patients was split into 10 smaller pools each containing 8208 individuals. Splitting the pool into 10 smaller pools allows us to run 10 independent simulations and average the results. From each pool, a cohort of 1000 patients was selected either at random or according to the selective recruitment protocol. At the end of each simulation, we fitted a Cox proportional hazards model and recorded which covariates were found to be statistically significant at α=0.05. These results were compared to a Cox model fitted to the full dataset of 82 089 patients. We found in our simulations that in the full dataset, 27 out of 30 covariates were found to be statistically significant. Of these 27, we found that, on average, nine were statistically significant using the selective recruitment protocol compared to an average of 6.8 when using a random protocol. An average of 0.4 and 0.2 of the three covariates which were not found to be significant in the full dataset were found to be significant in the selectively and randomly recruited cohorts respectively. The mean square difference between inferred model parameters in the selectively recruited cohorts and full dataset was 0.02 compared with 0.21 for randomly selected cohorts.

**FIGURE 5:**
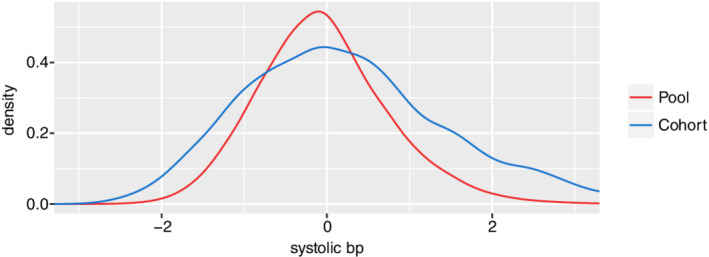
The empirical density of systolic blood pressure in a selectively recruited cohort of size 1000 compared to the pool of size 82 089 [Colour figure can be viewed at wileyonlinelibrary.com]

An obvious limitation here is that the parameters based on the full dataset are only estimators and not the true parameter values (which are unknown). Nevertheless, given the large size of the dataset (*N*=82 089) relative to the number of covariates (*d*=30), the estimated parameters will be reasonably accurate for the purposes of comparison to estimates based on a small subset (*n*=1000) of patients. The distribution of covariates within the selectively recruited cohorts was closer to a uniform distribution than the randomly selected cohorts. For each dichotomous covariate, we computed the ratio of the less frequent covariate value to the more frequent value. The median value of this ratio in the selectively recruited cohorts was 0.32 compared with 0.13 in the randomly selected cohorts. In Figure 5, the empirical cohort density of systolic blood pressure is plotted for one instance of a selectively recruited cohort and compared to the pool density. The covariate has a broader distribution than the pool. Further figures are available in Supplementary Figure 3. The characteristics of this selectively recruited cohort are compared with the full Caliber dataset in Table 1.

## 6 DISCUSSION

We have shown that preferential selection of a cohort with an informative distribution of covariates can lead to greater statistical power for a given sample size. In this article, informativeness is defined in terms of a covariate distribution that is as close to uniform as possible. We have shown that our selective recruitment protocol outperforms random selection in terms of power, sample size, and mean square error between true and inferred parameters in numerical simulations. Furthermore, we demonstrated the feasibility of our strategy by simulating realistic prospective observational studies using the CALIBER resource, an EHR with 82 089 patients. A similar study has previously been conducted in the U.S. and our results indicate that using EHR resources to selectively recruit patients would result in smaller sample size requirements.

Alternative measures of informativeness based on the posterior entropy and the expected decrease in prediction error have previously been investigated,^22^, ^35^ although such approaches are sensitive to the choice of statistical model. For instance, previous research found that in a logistic regression model or a proportional hazards model individuals with extreme covariate values are deemed most informative since effect sizes are implicitly assumed to be most pronounced in these individuals. Note that misspecification of the statistical model will in general lead to biased inference results, and this is a limitation of both selective recruitment and random recruitment strategies.

Researchers considering EHR based recruitment therefore have a number of recruitment strategies available. They could choose a randomly selected cohort, or a cohort with a close to uniform distribution of covariates, or preferentially recruit a cohort based on more sophisticated measures of informativeness such as those described above. Under all of these strategies, parameter estimates in a statistical model will converge toward the same values, but with varying degrees of statistical power. Preferential selection of informative cohorts has the potential to reduce the overall sample size requirements leading to more cost‐effective studies. On the other hand, a potential shortcoming is that a selectively recruited cohort may not be representative of the pool. A cohort that deviates substantially from the pool population may compromise the generalizability of the study, or limit the usefulness of the collected data for future research. The appropriateness of selective recruitment designs depends on striking an appropriate balance between the informativeness and representativeness of the cohort. The degree to which the cohort distribution deviates from the population distribution can be controlled in order to achieve an appropriate tradeoff between these competing considerations.

EHRs offer a potentially useful recruitment aid for clinical studies. A medical center could use a local database of patients in order to identify patients with a particular condition for the purposes of a study. National level EHRs could help to identify patients with rare conditions and help to form a cohort with a favorable composition. The techniques considered here may also be applicable to the recruitment of patients for clinical trials. It was previously shown that in trials with biomarkers it may be advantageous to select cohorts that have statistically desirable biomarker distributions.^22^, ^35^ We have restricted our present analysis to observational studies but an extension to randomized trials will be considered in future work. Another application of the protocol proposed here is to the cohort selection of a follow‐up study to a clinical trial. In such scenarios, a subset of patients are typically followed over a longer time period in order to acquire further evidence and monitor for adverse side effects. Here too, selective recruitment methods may be useful for selecting the maximally informative subset of individuals for the follow‐up study. We anticipate that in the future the prospect of leveraging EHRs to boost recruitment will become increasingly attractive.

## 7 CONCLUSION

EHRs present an opportunity to select a subset of individuals from a larger pool for the purposes of a clinical study. Rather than randomly selecting a cohort, preferentially composing a cohort with an informative covariate distribution may offer increased statistical power, lower mean square error, and smaller sample size requirements without compromising the type I error rate.

## Supporting information

Data S1: Supporting Information
