# Supplementary material for: Selective recruitment designs for improving observational studies using electronic health records
Source: Stat Med. 2020 Jun 10;39(19):2556–67. doi: 10.1002/sim.8556 (PMC8432147; doi:10.1002/sim.8556)
Supplement: Supplementary file 1 — Data S1: Supporting Information [file SIM-39-2556-s001.pdf]

Supplementary material: selective recruitment designs for improving observational studies using electronic health records

James E. Barrett*†[1], Aylin Cakiroglu†[2], Catey Bunce[3], Anoop Shah[4,5,6], and Spiros Denaxas[4,5]

[1] *Cancer Cell Biology & Imaging, King's College London, London, SE1 1UL, U.K.*
[2] *The Francis Crick Institute, London, NW1 1AT, U.K.*
[3] *Division of Health & Social Care Research, King's College London, London, SE1 1UL, U.K.*
[4] *UCL Institute of Health Informatics, University College London, Gower Street, London WC1E 6BT, U.K.*
[5] *Health Data Research U.K., London, U.K.*
[6] *University College London Hospitals NHS Trust, 250 Euston Road, London NW1 2PG, U.K.*

September 19, 2019

1 Numerical simulations

1.1 Type I error rate

A pool of $N = 10,000$ individuals with two binary covariates was generated from the distribution shown in Figure 2 (a) of the main text. Binary outcomes $y = \pm 1$ were generated according to a logistic regression model $p(y = +1|\mathbf{x}) = 1/(1 + \exp(-w_0 - \mathbf{w} \cdot \mathbf{x}))$. The parameters were set to $w_0 = -1/6$ and $\mathbf{w} = (1/3, 0)$. Cohorts of size n were selecting around to the marginally balanced, jointly balanced, and random selection protocols. For each cohort a logistic regression model was fitted. The null hypothesis of no association between covariates and outcomes is true for the second component of $\mathbf{w}$. In order to estimate the type I error rate we calculated the proportion of times this parameter was inferred to be significant at a 0.05 significance level. The results are shown in Supplementary Figure 1. The type I error rate is well controlled under all selection protocols.

1.2 Unmeasured covariates

A pool of $N = 10,000$ individuals with two binary covariates was generated from the distribution shown in Figure 2 (a) of the main text. Binary outcomes $y = \pm 1$ were generated according to a logistic regression model $p(y = +1|\mathbf{x}) = 1/(1 + \exp(-w_0 - \mathbf{w} \cdot \mathbf{x}))$. The parameters were set to $w_0 = -1/6$ and $\mathbf{w} = (= -1/3, 1/4)$. After the outcomes had been generated the second covariate

*Contact: james.barrett@kcl.ac.uk
†These authors contributed equally to this work.

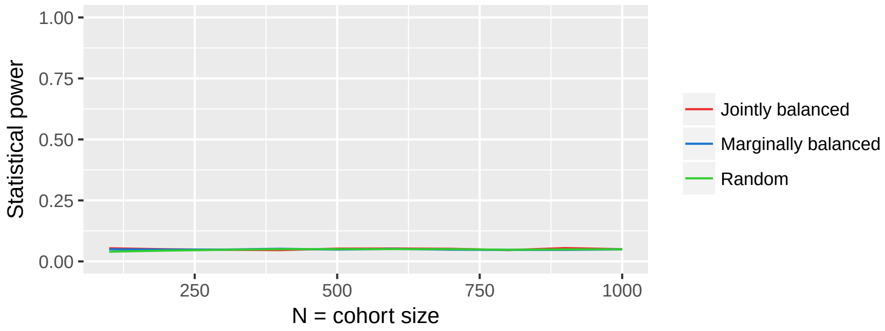

Figure 1: Type I error rate as a function of cohort size for the case of two binary covariates.

was removed from the pool (that is, it was an unmeasured covariate). The marginal distribution of the remaining covariate was $p(x_1 = 1) = 0.75$ and $p(x_1 = -1) = 0.25$.

Cohorts of size n were selected from the pool according to the jointly balanced, and random selection protocols. For each cohort a logistic regression model was fitted. The mean square error between the inferred and true parameter w_1 is shown in Supplementary Figure 2. The unmeasured covariate introduces a bias into the parameter estimate but this is the same for both types of recruitment protocols.

For comparison a second experiment was run in which a pool of $N = 10,000$ individuals with a single binary covariate were generated. A logistic regression model with $w_0 = -1/6$ and $w = -1/3$ was used to generate outcomes. Cohorts of size n were selected as above. The mean square error between inferred and true parameter values is also plotted in Supplementary Figure 2. Due to the absence of unmeasured covariates there is no bias in this case.

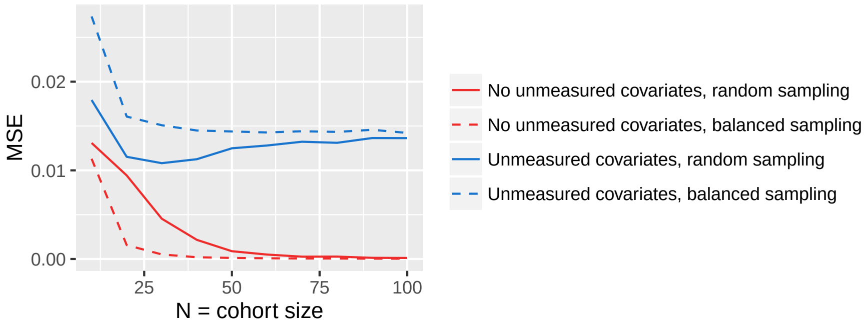

Figure 2: Mean square error in the presence and absence of unmeasured covariates.

2 The CALIBER dataset

2.1 Study population

We selected patients from 225 primary care practices registered between January 1997, and March 2010, who fulfilled the following criteria which are aligned with previous work described in Rapsomaniki et al. (2014):

- Are 18 years or older at study start date or turn 18 during the study period time
-
  - Stable angina, defined by Read codes in CPRD for angina diagnosis, positive ischaemia tests, coronary angiogram results recorded or repeat prescriptions for nitrates, or in HES by hospitalisations with a primary spell diagnosis ICD10 code I20.1, I20.8, or I20.9
  - Myocardial Infarction non-fatal, as defined by Read codes in CPRD or ICD10 I21?I22 as the primary diagnosis in HES
  - had other coronary artery disease (CHD) in CPRD/HES data, i.e. coronary artery bypass graft (CABG), or percutaneous coronary intervention (PCI)
  - Unstable angina, defined by Read codes in CPRD or hospital admission with ICD10 code I20.0

Patients with prior acute events were classified as stable if they survived longer than 6 months after the acute event, and only entered the cohort at this point following definitions in Rapsomaniki et al. (2014). Patients entered on the date of the first myocardial infarction after study eligibility (minimum of one year after registration in a contributing GP practice).

2.2 Endpoints

The primary endpoint was all-cause mortality as defined in ONS or CPRD. Patients were censored at the earliest date among death date, relocation to a new primary care practice, or study end date (25 March 2010) following definitions in Rapsomaniki et al. (2014).

2.3 Imputation

Multiple imputation was implemented using multivariate imputation by chained equations in the R package mice van Buuren and Groothuis-Oudshoorn (2011). Imputation models were estimated separately for men and women using all 115,305 patients before exclusion criteria were applied (MI or death before study eligibility) and included:

- All the baseline covariates used in the main analysis (age, diabetes, smoking, systolic blood pressure, diastolic blood pressure, total cholesterol, HDL cholesterol, body mass index, serum creatinine, haemoglobin, total white blood cell count, CABG or PCI surgery within 6 months prior to study entry, abdominal aortic aneurysm prior to study entry, index of multiple deprivation, ethnicity, hypertension diagnosis or medication prior to study entry, use of long acting nitrates prior to study entry, diabetes diagnosis prior to study entry, peripheral arterial disease prior to study entry, and quadratic age.

- Prior (between 1 and 2 years before study entry) and post (between 0 and 2 years after study entry) averages of continuous main analysis covariates and other measurements not in the main analysis (Hba1c, eFGR-CKDEPI, lymphocyte counts, neutrophil counts, eosinophil counts, monocyte counts, basophil counts, platelet counts, pulse pressure).
- Coexisting medical conditions (history of heart attack, depression, anxiety disorder, cancer, renal disease, liver disease, chronic obstructive pulmonary disease, atrial fibrillation, or stroke prior to study entry)
- The Nelson-Aalen hazard and the event status for each endpoint analyzed in the data.

Since many of the continuous variables were non-normally distributed, we log-transformed all continuous variables for imputation and exponentiated back to their original scale for analysis. Only one multiply imputed dataset was generated since any imputation errors are not expected to have a significant effect on our analyses in respect to the comparison of different designs. The distributions of observed and imputed values of all variables followed similar distributions indicating the plausibility of the imputation.

References

Eleni Rapsomaniki, Anoop Shah, Pablo Perel, Spiros Denaxas, Julie George, Owen Nicholas, Ruzan Udumyan, Gene Solomon Feder, Aroon D. Hingorani, Adam Timmis, Liam Smeeth, and Harry Hemingway. Prognostic models for stable coronary artery disease based on electronic health record cohort of 102 023 patients. *European Heart Journal*, 35(13):844–852, 2014. doi: 10.1093/eurheartj/eht533. URL `+http://dx.doi.org/10.1093/eurheartj/eht533`.

Stef van Buuren and Karin Groothuis-Oudshoorn. mice: Multivariate imputation by chained equations in r. *Journal of Statistical Software*, 45(3):1–67, 2011. URL `http://www.jstatsoft.org/v45/i03/`.

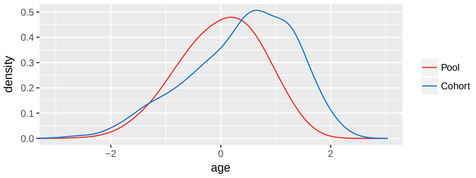

(a)

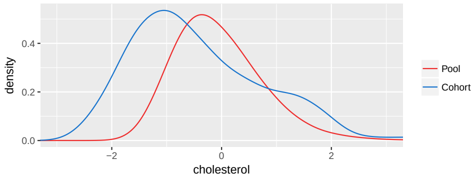

(b)

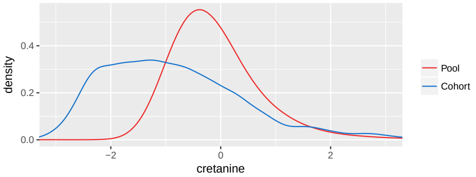

(c)

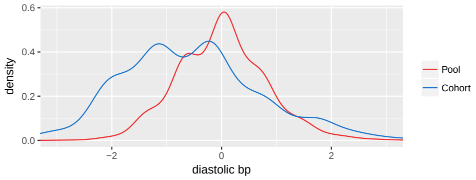

(d)

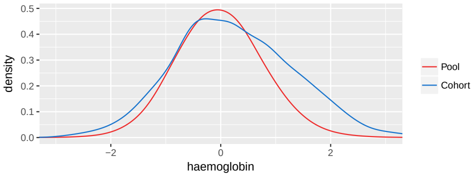

(e)

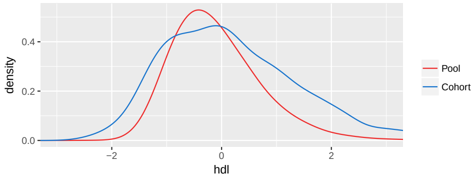

(f)

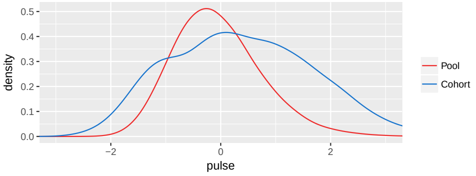

(g)

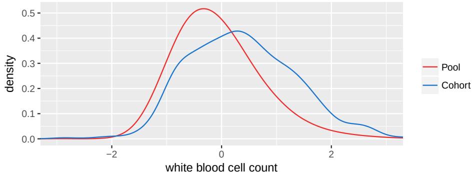

(h)

Figure 3: Empirical densities of continuous covariates in a selectively recruited cohort ($N = 1,000$) and the pool ($N = 82,089$).

Covariate	Inferred $\boldsymbol{\beta}$	Lower CI	Upper CI	p-value
age	1.274	1.252	1.295	0
gender	0.102	0.084	0.121	1.883265e-27
deprived	-0.058	-0.076	-0.039	8.060814e-10
CHD	0.198	0.179	0.216	1.962481e-97
UA	0.042	0.024	0.061	7.265859e-06
NSTEMI	0.118	0.1	0.137	4.082319e-36
STEMI	-0.01	-0.028	0.009	0.3110636
PCI	-0.067	-0.085	-0.048	1.220014e-12
CABG	-0.134	-0.152	-0.115	1.291749e-45
MI	0.03	0.012	0.049	0.001271308
nitrates	0.045	0.026	0.063	2.272479e-06
smoke	0.12	0.102	0.139	3.595763e-37
hypertension	0.022	0.004	0.041	0.01787763
diabetes	0.144	0.125	0.162	1.925671e-52
chol	0.001	-0.023	0.024	0.9572574
hdl	0.003	-0.02	0.025	0.826079
heart.failure	0.248	0.229	0.266	4.505281e-152
PAD	0.169	0.151	0.187	8.125267e-72
AF	0.16	0.141	0.178	2.189484e-64
stroke	0.224	0.206	0.243	4.102543e-125
renal	0.174	0.155	0.192	8.507037e-76
COPD	0.065	0.046	0.083	6.082837e-12
cancer	0.229	0.211	0.248	1.038268e-130
liver	0.48	0.462	0.499	0
depression	0.06	0.042	0.079	1.906276e-10
anxiety	-0.065	-0.084	-0.047	3.798424e-12
crea	0.052	0.034	0.069	3.374309e-09
wbc	0.056	0.035	0.077	1.842707e-07
haemo	-0.092	-0.114	-0.07	9.550895e-17

Table 1: Inferred parameters from a Cox proportional hazards model applied to the full CALIBER dataset ($N = 82,089$).
